# A Comprehensive Model for Cost-Benefit Analysis of Telehealth Awareness and Digital Literacy Training

**DOI:** 10.1093/geroni/igaf122.1901

**Published:** 2025-12-31

**Authors:** Paul Freddolino, Ying (Jessica) Cao

**Affiliations:** Michigan State University, East Lansing, Michigan, United States; University of Wisconsin–Madison, Madison, Wisconsin, United States

## Abstract

Even before the COVID-19 pandemic telehealth use was increasing globally. The pandemic supercharged this trend due to prohibitions against in-person services. Practitioners and patients found that telehealth provided increased access to primary and specialty care, overcame transportation barriers, permitted better-coordinated chronic condition management, and potentially reduced healthcare costs. In the post-pandemic world telehealth use is stabilized at around 20% of all outpatient encounters. However, many older adults still face barriers, particularly limited IT literacy. Direct care workers (DCWs) who provide personal care and support can help address these barriers if trained. While some DCWs receive digital training, direct evidence of cost and benefits of such digital health literacy training is still limited. This presentation aims to fill this knowledge gap based on insights from two community projects. The first utilizes several coaching models to enhance digital literacy including telehealth awareness for older adults. The second provides similar skill-building to DCWs of older adults with the intent that DCWs will share this new knowledge with their clients and family caregivers. We will present a comprehensive model for assessing the costs and benefits of telehealth awareness and digital literacy training for DCWs through the standpoint of four stakeholder groups: direct care workers, patients, unpaid family caregivers, and providers/employers of DCWs (e.g., hospitals). For each group the benefit model explores functional value/benefit, economic value, social value, and emotional value. The cost model includes hiring, training, workload, cognitive burden, and other factors. Potential measurement strategies for model components will also be reviewed.

